# Potential Roles of Acute Phase Proteins in Cancer: Why Do Cancer Cells Produce or Take Up Exogenous Acute Phase Protein Alpha1-Antitrypsin?

**DOI:** 10.3389/fonc.2021.622076

**Published:** 2021-02-19

**Authors:** Sabina Janciauskiene, Sabine Wrenger, Steffen Günzel, Anna Ricarda Gründing, Heiko Golpon, Tobias Welte

**Affiliations:** Biomedical Research in Endstage and Obstructive Lung Disease Hannover (BREATH), Member of the German Center for Lung Research (DZL), Department of Respiratory Medicine, Hannover Medical School, Hannover, Germany

**Keywords:** cancer cells, alpha1-antitrypsin, acute phase proteins, apoptosis, autophagy, cancer microenvironment

## Abstract

An association between acute-phase proteins (APPs) and cancer has long been established and there are numerous reports correlating altered levels and/or molecular forms of APPs with different types of cancers. Many authors have shown a positive correlation between high levels of APPs, like alpha1-antitrypsin (AAT), and unfavorable clinical outcome in cancers. Conversely, others proposed that high levels of APPs are probably just a part of nonspecific inflammatory response to cancer development. However, this might not be always true, because many cancerous cells produce or take up exogenous APPs. What is the biological significance of this and what benefit do cancer cells have from these proteins remains largely unknown. Recent data revealed that some APPs, including AAT, are able to enhance cancer cell resistance against anticancer drug-induced apoptosis and autophagy. In this review, we specifically discuss our own findings and controversies in the literature regarding the role of AAT in cancer.

## Introduction

The relationship between inflammation, immunity and cancer is widely accepted. The cancer-related inflammatory responses can occur due to the chemotherapy as well as due to the tumor cell migration, invasion, activation of anti-apoptotic signaling pathways, and metastasis (1). Cancer-caused inflammation may result in the activation of transcription factors like nuclear factor-κB, signal transducer and activator of transcription 3, and hypoxia-inducible factor 1α, which further enhances inflammatory response. Accordingly, markers of systemic inflammation, including C-reactive protein (CRP), albumin, neutrophils, lymphocytes, neutrophil-to-lymphocyte ratio, platelet-to-lymphocyte ratio, and cytokines have been shown to be altered in patients with various cancers (2). These changes in local and systemic inflammatory mediators can induce epigenetic changes that favor tumor initiation, and create a milieu to either enhance or suppress cancer development (3).

The hallmarks of cancer-related environment include immune cells, stromal cells (including myofibroblasts), and inflammatory mediators, tissue remodeling and angiogenesis factors, similar like those seen in chronic inflammatory conditions or in tissue repair. Under the influence of cytokines and chemokines, liver but also other cells, like endothelial and epithelial cells, produce acute phase proteins (APPs). Experimental observations suggest that various cytokine combinations may have additive, synergistic, or inhibitory effects on the production of individual APPs (4). On the other hand, APPs *per se* modulate inflammatory reaction, as can be illustrated by the CRP example. First, pro-inflammatory cytokines/chemokines, like IL-6, TNFα, and IL-8 induce the production and release of CRP (5), but thereafter CRP itself increases the release of these pro-inflammatory mediators. In line, the infusion of recombinant human CRP into healthy volunteers was found to result in a significant induction of serum levels of the IL-6 and IL-8 as well as amyloid A, phospholipase A, and coagulation markers (6). These observations support a notion that CRP, but also other APPs, are not only markers of inflammation. The functions of APPs are important in trapping of microorganisms and their products, in activating the complement system, in neutralizing enzymes, scavenging free hemoglobin and radicals, and in modulating the host’s immune response. Thus, APPs actively participate in the development and resolution of inflammation, and the overall profiles of APPs seem to depend on the nature of the initial inflammatory event, and how this event induces a systemic protein/cytokine response.

Elevated levels of APPs in patients with a variety of cancers were suggested to serve as prognostic or diagnostic markers in the context of clinical examinations. Interestingly, many types of cancer can express but also take up exogenous APPs, which may influence drug resistance, cancer progression and metastasis (7). For example, recent data have shown that the property of cancer cells to produce serum amyloid A enhances their resistance to T-cell immunity due to the activation of immunosuppressive granulocytes (8, 9).

The relationship between tumorigenesis and altered levels of circulating or tumor-associated APPs, such as haptoglobin, ceruloplasmin, CRP, alpha-2 macroglobulin, alpha-1-acid glycoprotein, plasminogen activator inhibitor-1 and alpha-1-antitrypsin has been reported (**Table 1**).

**Table 1:** Acute phase proteins in cancer.

Acute phase protein	Function	Cancer type	Reference
Haptoglobin (Hp) 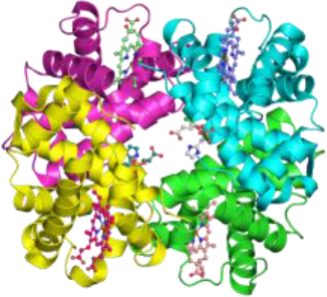	– Iron-containing oxygen-transport metalloprotein – Scavenger for free hemoglobin – Inhibitor of prostaglandin production – Regulator of leukocyte recruitment and cytokine release – Prognostic biomarker for NSCLC	Increased in ovarian, colorectal, pancreatic, breast and lung cancer	(10–14)
Serum amyloid A (SAA) 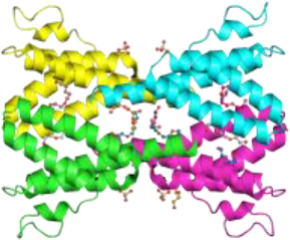	– Apolipoprotein associated with high-density lipoprotein – Regulator of cell-cell communication, inflammatory, immunologic, neoplastic and protective pathways – Prognostic biomarker for solid tumors	Increased in gastric cancer, colorectal cancer, NSCLC, melanoma, renal cancer, neuroblastoma	(15–20)
Ceruloplasmin (Cp) 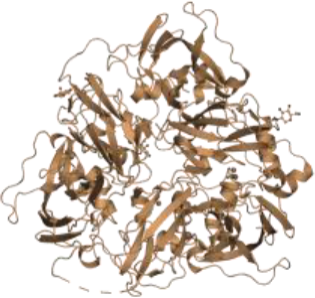	– Major copper transport protein, oxidase – Protector against oxidative stress – Potential cancer biomarker	Increased in hepatocellular carcinoma, breast cancer, cervical cancer, bile duct cancer	(21–24)
Alpha-1-acid glycoprotein (AGP, orosomucoid) 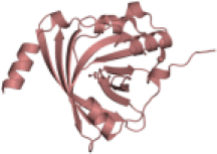	–Carrier of basic and neutrally charged lipophilic compounds – Potential biomarker in breast cancer	Increased in plasma and ascites of cancer patients with peritoneal carcinomatosis, breast cancer	(7, 25)
Plasminogen activator inhibitor 1 (PAI-1) 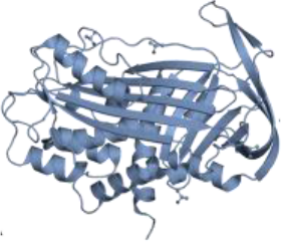	– Serine protease inhibitor – Inhibitor of tissue plasminogen activator and urokinase, – Pro-tumorigenic and anti-apoptotic – Stimulator of angiogenesis	Increased in breast, ovarian, bladder, colon cancer and NSCLC	(26–30)
C-reactive protein (CRP) 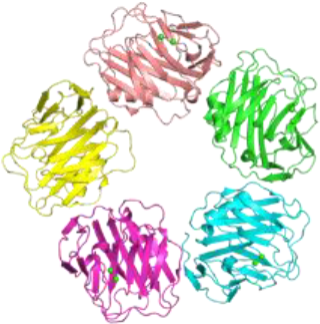	– Modulator of inflammatory processes and host responses to infection including complement pathway, apoptosis, phagocytosis, nitric oxide release, and production of cytokines – Prognostic marker for solid tumors including NSCLC	Increased in stomach, pancreas, colorectal, esophageal, ovarian, renal, breast cancer and NSCLC, melanoma, neuroblastoma	(5, 31, 32)
Alpha-2-macroglobulin (AMG) 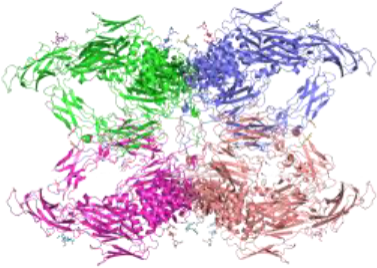	– Broad spectrum protease inhibitor – Carrier of growth factors and cytokines – Antioxidant, anti-fibrotic and anti-inflammatory – Protects against radiation induced cell damage – Suggested biomarker for the diagnosis of B-cell acute lymphoblastic leukemia	Decreased levels in more progressed prostate cancer; Increased levels in acute lymphoblastic leukemia	(33–35)
Alpha1-Antitrypsin (AAT) 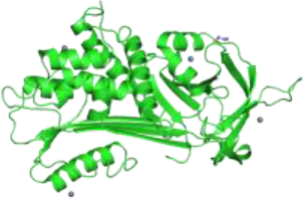	– Broad spectrum protease inhibitor – Anti-inflammatory and immunomodulatory – Regulator of cell adhesion, migration, invasion, proliferation and angiogenesis – Anti-apoptotic – Prognostic marker	Increased serum levels in NSCLC, pancreas, prostate, cervix, ovary, breast, larynx and other carcinomas	(36–52)

NSCLC, non-small cell lung cancer.

Single APPs are used as biomarkers, especially for cancer prognosis and diagnosis of complications to anticancer treatments. However, clinical studies show that the inflammatory phenotype (i.e., the APPs/cytokine profile) differs between patients not only with different malignancies but also with the same malignant disease (53). Therefore, we and other scientists think that APPs may become more valuable biomarkers if used in combinations or used with other acute phase markers, as a part of an acute phase profile. So far, the most commonly investigated prognostic markers were CRP, albumin, and the CRP: albumin ratio.

Regarding single APPs, an extensive literature supports the pro-tumorigenic activity of plasminogen activator inhibitor 1 (PAI-1, Serpin E1) (54). Clinical studies have shown that higher expression of PAI-1 positively correlates with a poor clinical outcome in patients with breast, ovarian, bladder, colon and non-small cell lung cancers (NSCLC) (26). A number of experimental studies described PAI-1 as an anti-apoptotic protein and a stimulator of angiogenesis (27, 28). On the other hand, employment of small molecule or antibody inhibitors of PAI-1 so far provided no evidence that inhibition of PAI-1 could have any therapeutic effect in cancer patients (29, 30). Likewise, another protease inhibitor, alpha1-antitrypsin (AAT, Serpin A1) has been identified as a prognostic marker of tumor recurrence and prognosis. Our recent results obtained from more than 300 patients with NSCLC revealed that higher serum levels of AAT are prognostic for the patient’s worse outcome (36). Nevertheless, studies regarding the role of AAT in lung cancer are contradictory. Some demonstrate a direct relationship between high levels of AAT and the risk of lung cancer (36, 37) while opposite, others associate genetic AAT deficiency with an increased risk of lung cancer development (55). In this review, we specifically discuss our own findings and controversies in the literature regarding the role of AAT in lung cancer. Insights gained into the action of AAT towards lung cancer cells could be exploited for the future understanding of APPs’ role in tumorigenesis.

## Overexpression or Deficiency of Human AAT, Which One Is Related to Tumorigenesis

### AAT Functions Beyond Serine Protease Inhibition

Human alpha1-antitrypsin (AAT), a member of the serpin (serine protease inhibitor) superfamily, is an acute phase glycoprotein and the best recognized inhibitor of neutrophil elastase. Various studies show that AAT also inhibits the activity of metalloproteases and caspases that play an essential role in cell apoptosis. AAT is a product of *SERPINA1* gene mainly expressed by hepatocytes and monocytes/macrophages. Its expression is regulated by interleukin 6-type cytokines. Both genetic and environmental factors influence an individual’s basal level of AAT, and thus circulating AAT in apparently healthy people can vary from 1 to 2 g/L (56).

Experimental and clinical studies provide evidence that AAT expresses broad anti-inflammatory and immunomodulatory properties, some of which unrelated to protease inhibition. For example, AAT interacts and builds complexes with various inflammation-related ligands including chemokines IL-8 and LTB4, complement factors, and heat-shock proteins (57–60). Moreover, AAT regulates cellular adhesion, chemotaxis, and wound healing and modulates signaling pathways to promote cell proliferation and angiogenesis (61).

### AAT Favors Cancer Development

During chronic inflammation, which is a driving force in cancer development, increased levels and functional activity of AAT can rescue not only normal but also cancer cells. Indeed, AAT seems to be involved in the metastatic outgrowth of various cancer types including ovarian, cervical, colorectal, breast, and lung adenocarcinomas (38–42). Elevated plasma levels of AAT have been reported in patients with Hodgkin’s lymphoma, pancreas, prostate, cervix, ovary, breast, larynx, colorectal, and other carcinomas, and proposed to be useful as prognostic factors (43–52). In breast cancer a high level of AAT has been associated with poor clinical prognosis (62). Furthermore, AAT seems to be directly involved in metastasis of ovarian, cervical, colorectal, breast, and lung adenocarcinomas (39, 40). Given an increasing body of research in this area, with pro-tumorigenic claims about AAT, we and others raised the hypothesis that individuals with inherited AAT deficiency might have a lower risk of developing cancer.

### AAT Deficiency—Protects or Predisposes to Cancer Development

The deficiency, defined as an AAT blood level less than 35% of the mean expected value, is usually associated with Z allele (point mutation Lys342Glu), and less frequently with combinations of S (Lys264Val) or null alleles (63). Severe AAT deficiency predisposes to early onset of chronic obstructive pulmonary disease with emphysema, to liver cirrhosis at any age, neutrophilic panniculitis, systemic vasculitis, and possibly other inflammatory disease risks (64, 65). Severe AAT deficiency is a risk factor of developing hepatocellular carcinomas because of the damage of hepatocytes caused by retained intracellular polymers of mutant AAT protein, and an inappropriate hepatocellular regeneration. Data from the Swedish National AAT Deficiency Register revealed hepatocellular cancer diagnosis in 32 (2%) out of 1,595 individuals with ZZ AAT deficiency (66). Nevertheless, a risk of hepatocellular carcinoma in AAT deficiency is difficult to quantify because of a global variation in AAT genetics and the incidences of liver cancer (67).

In contrast, there are other reports showing that AAT deficiency people have lower or no increase in risk of developing lung and probably other types of cancers. For example, results from a cross-sectional survey study conducted among 720 AAT deficient people of the Alpha-1 Foundation Research Registry at the Medical University of South Carolina have shown that only 8 (1.1%) of participants have a diagnosis of lung cancer. In line, the comparison of 1585 ZZ AAT deficient subjects from Swedish AAT Deficiency Register and 5,999 population-based controls showed that death due to cancer in general was lower among ZZ individuals compared to the controls, i.e. 11% versus 33%. Pulmonary carcinoma accounted for 1% of the causes of death in ZZ individuals and 4% in the controls (68). There are ongoing clinical studies designed to downregulate endogenous AAT expression within hepatocytes by using small-interfering RNA (siRNA) based drugs (69–71). These studies aim to cure/prevent liver disease in individuals with inherited AAT deficiency caused by intrahepatic accumulation of mutant AAT (63). Hypothetically, this approach of a temporal elimination of AAT protein synthesis might be helpful in the field of cancer and warrants further investigations.

### Therapy With Human Plasma AAT Shows No Risk for Cancer Development

It is important to point out that the therapy with human plasma AAT used for over three decades to treat patients with inherited AAT deficiency-related emphysema does not rise tumor development rates. In fact, if untreated, individuals with inherited AAT deficiency seem to have higher risk of developing lung (72), gastrointestinal (73), and liver cancers (74). Likewise, few studies in patients with colorectal and gastric cancer found less AAT in both tissues and serum than in normal counterparts (75, 76). Therefore, one needs carefully consider differences between non-tumor and tumor-related environmental settings. The involvement of AAT in tumorigenesis may strongly depend on cancer cell properties as well as on the concentration and molecular forms of AAT, which are influenced by genetic and microenvironmental factors. For example in the B16-F10 lung metastasis mice model, Guttman and co-authors provided experimental evidence that NK cell-sensitive tumors are unaffected whereas CD8^+^ T cell-sensitive tumors can be significantly inhibited by the treatment with human AAT (77). It is also important to notify, that during inflammatory conditions AAT can undergo post-translational modifications like S-nitrosylation on its single surface cysteine residue, forming S-NO-AAT, a reducer of tumor cell viability (78).

### Role of AAT in Non-Small Cell Lung Cancer

Non-small cell lung cancer (NSCLC) is one of the most common lung cancers worldwide, and about 70% of patients with this cancer present with advanced or metastatic disease at the time of diagnosis (79). Hitherto, there are only limited studies addressing the role of AAT in lung cancer (36, 37, 55). Therefore, to gain further insights into this matter, Schwarz et al. investigated the effects of extracellular AAT on NSCLC cell behavior, *in vitro* (80). By comparing cancer cells grown in a regular medium versus medium supplemented with AAT, authors found that in the presence of physiological concentrations (0.5–1 mg/ml) of AAT cells acquire better pro-tumorigenic properties, and second that AAT strongly enhances cancer cell resistance against staurosporine-induced toxicity (80).

**Figure 1:**
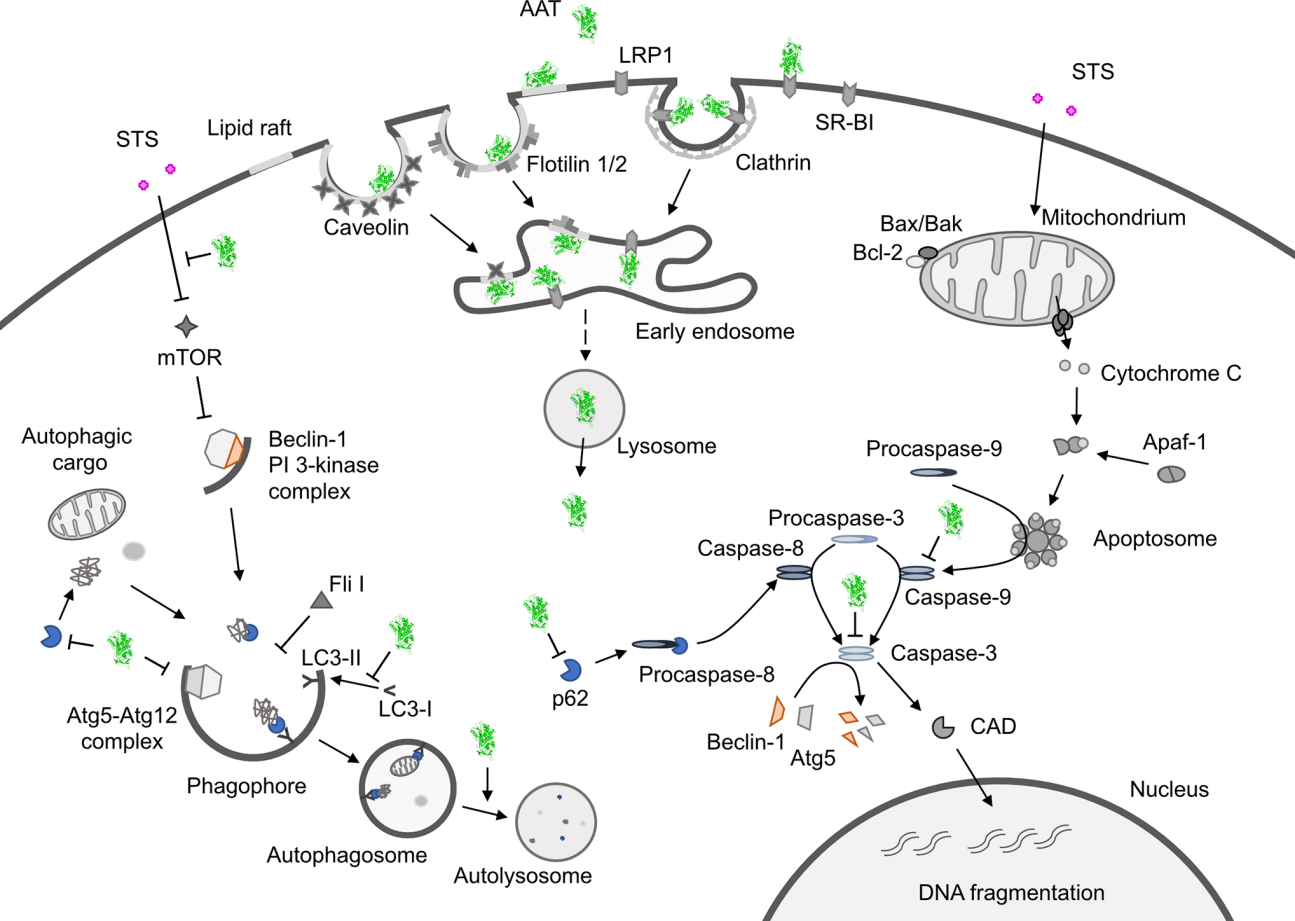
Hypothetical model showing how alpha1-antitrypsin (AAT) may affect staurosporine (STS)-induced apoptosis and autophagy in NSCLC cells. Intracellular entry of AAT occurs constitutively based on lipid raft independent pathways, namely clathrin-mediated, or lipid rafts-dependent, which include caveolae- and flotilin-dependent endocytosis. The AAT could be sequestered into clathrin-coated pits by binding to LRP1 (low-density lipoprotein receptor related protein 1) and SR-BI (scavenger receptor class b type I). Alternatively, AAT can associate with caveolin or flotilin 1/2-postive lipid rafts. All entry pathways might generate AAT-containing vesicles fusing with early endosomes and lysosomes from which—by unknown mechanisms - AAT escapes into the cytoplasm (*green structure*). There, AAT could affect cancer cell responses to cytotoxic drugs, like STS (staurosporine). STS activates autophagy by inhibiting mTOR (mammalian target of rapamycin), which allows Beclin-1/PI 3-kinase complex formation, increases LC3-II/LC3-I ratio, p62 degradation in autolysosomes and downregulation of Fli I (Flightless I), a controller of p62-LC3 interaction. Indeed, AAT seems to block different steps required for STS to induce autophagy. Potentially, AAT may also interfere with Atg5-Atg12 complex required for the formation of autophagosomes or recruitment of LC-3 to autophagosomes (*green structures*). As an inducer of apoptosis, STS acts through the activation of the mitochondrial apoptotic pathway. STS causes the aggregation of Bax/Bak and the release of cytochrome c that binds to Apaf-1 (apoptotic protease-activating factor 1) allowing apoptosome assembly and the recruitment of procaspase-9 to the apoptosome. In this scenario AAT might inhibit activation of procaspases-8, -9, -3 preventing Beclin-1/Atg5 degradation and activation of CAD (caspase-activated DNase). A crosstalk between STS-induced autophagy and apoptosis is in part mediated by p62 and beclin-1. Finding that AAT by itself strongly reduces p62 levels but prevents p62 reduction in STS-treated cells suggests that cancer cells utilize AAT to regulate apoptosis and autophagy dependent on the situation. Under basal conditions, AAT as a reducer of p62 protein levels (also important for the activation of procaspase-8), might activate autophagy as a cytoprotective pathway. In the setting when cancer cells face pro-apoptotic activation, autophagy inhibition may become a strategy to escape apoptosis.

Staurosporine is a nonspecific protein kinase inhibitor derived from *Streptomyces staurosporeus* that can trigger cancer cell death through activation of both, apoptosis and autophagy pathways. Several lines of evidence indicate a cross-talk or mutual function between autophagy and apoptosis (81). As an inducer of apoptosis, staurosporine specifically acts through the activation of the mitochondrial apoptotic pathway, which is mainly controlled by Bak and Bax proteins (82, 83). When activated, these proteins can form pores on the mitochondrial outer membrane causing cytochrome c release that binds to apoptotic protease activating factor 1 (Apaf-1) and induces its oligomerization, known as the apoptosome formation. The apoptosome promotes cleavage of procaspase-9 into active caspase-9 which in turn activates caspases-3 and -7 to execute the final steps of apoptosis (84) (**Figure 1**).

## AAT Inhibits Staurosporine-Induced NSCLC Cell Apoptosis

According to the results from Schwarz et al., staurosporine-induced NSCLC cell apoptosis was completely hampered in the presence of AAT. More detailed investigations revealed that AAT prevented the cleavage of procaspase-3, a precursor of caspase-3 which is one of the most deleterious executioner caspases in apoptosis pathway (80). As yet, the anti-apoptotic effects of AAT have been mostly attributed to the direct inhibition of caspase-3 activity. Investigators reported that exogenous AAT reduces the activity of caspase-3 in ß-cells, lung endothelial and epithelial cells, cardiomyocytes and neutrophils (85–88), and that AAT inhibits active caspase-3 by forming an AAT-caspase-3-complex (89). In cell-free assays, AAT was also demonstrated to inhibit other executioner caspases, especially caspases-6 and -7, but not the initiator caspases, like caspase-9 (90). However, the results presented by Schwarz et al., indicate that protective effects of AAT against staurosporine-induced NSCLC cells apoptosis might be earlier in the apoptosis pathway than the inhibition of executioner caspases. In concordance, a recent study has shown that AAT significantly suppresses cytokine-induced cleavage of procaspase-9 in human islets (91).

## Crosstalk Between Apoptosis and Autophagy

Several autophagy proteins are substrates for caspase-induced apoptosis. For example, caspase-3 and -9-mediated cleavage of Atg5 (E2-like enzyme is required for the formation of autophagosomes) and beclin-1 (implicated in the autophagic programmed cell death) switches autophagy to apoptosis (92). Atg5 has also been found to be cleaved by calpain and translocate to the mitochondria to contribute to the release of cytochrome c. Previous finding that AAT inhibits calpain activity in human neutrophils (93) allows speculating that AAT may protect cancer cells *via* inhibition of other proteases than caspases.

## AAT Inhibits Staurosporine-Induced Autophagy in NSCLC Cells

The mechanism(s) by which AAT helps lung cancer cells to acquire resistance towards staurosporine-induced apoptosis can also involve a shift of the balance towards anti-apoptotic proteins. As a matter of fact, the overexpression of AAT protein in lung cancer cell lines resulted in increased Bcl-2 and decreased beclin-1 levels (94). Thus, AAT may promote cancer cell survival by increasing Bcl-2, an anti-apoptotic, membrane associated protein (95) and by inhibiting beclin-1-dependent autophagy and apoptosis (96–99).

As already mentioned above, similarly to other anticancer agents, staurosporine can induce cancer cell death by simultaneous activation of apoptosis and autophagy (100). Autophagy may serve as a backup process for apoptosis—to enhance and/or contribute to the apoptosis (101–103). Autophagy involves the formation of double-membraned structures known as autophagosomes responsible for the engulfment of cargoes that are subsequently degraded after fusion with lysosomes (104). Autophagosome formation involves a set of proteins named Atg (autophagy-related) that orchestrate autophagosome initiation and biogenesis. Currently, the light chain 3 (LC3) is considered as one of the best autophagosome markers because the amount of LC3-II reflects the number of autophagosomes (105). Again, the LC3-II amount as well as LC3-II/LC3-I ratio not necessarily mirrors the autophagic activity, since the amount of LC3-II might increase not only due to autophagy activation but also due to the inhibition of autophagosome degradation. Therefore, for monitoring autophagic activity is used p62 (known as sequestosome-1), autophagic substrate receptor that is constantly being degraded by autophagy (106, 107). The reduced phosphorylation of mTOR is one more factor well reflecting the activation of autophagic flux (108). Results by Schwarz et al. confirmed that staurosporine added to NSCLC cells activates autophagy as measured by increase in LC3-II/LC3-I ratio, reduced mTOR activity as shown by dephosphorylated/less phosphorylated ribosomal protein S6 (RP-S6), degradation of p62 and downregulation of Flightless I, controlling the binding activity of p62 to LC3 (80). Clearly, in the presence of AAT all above mentioned markers of autophagy activation were unaffected by staurosporine.

## Potential Pro-Tumorigenic Functions of AAT

Because autophagy can promote caspase-independent or caspase-dependent cell death (109–112), a major remaining question is whether effect of AAT on autophagy is independent or dependent of apoptosis. We would like to pay particular attention to the finding that, although AAT significantly prevented the reduction in p62 levels in staurosporine-treated NSCLC cancer cells, AAT by itself strongly reduced p62 protein levels as compared to non-treated controls. p62 is a regulator of selective autophagy (113) and recruiter of caspase-8 on autophagic membranes, which establishes a crosstalk between autophagy and apoptosis (114). Thus, the way by which cancer cells utilize AAT to regulate apoptosis and autophagy, probably is a context dependent. Under basal conditions, autophagy as a cyto-protective pathway can be activated by exogenous AAT whereas in the setting when cancer cells face pro-apoptotic activation, autophagy inhibition by AAT may become a strategy to enhance survival. In fact, earlier Shapira et al. reported that intracellularly synthesized AAT prevents autophagic cell death and that exogenous AAT added to the cells prior to the induction of autophagy by tamoxifen reduces autophagy and cell death (115).

One cannot exclude that AAT may also facilitate cell survival through other mechanisms than modulation of autophagy. For example, study by Seung-Hee Chang et al., found that in AAT-overexpressing L132 cancer cells, the expression of manganese superoxide dismutase (SOD2), a tumor suppressive protein acting *via* inhibition of cell proliferation and induction of apoptosis (116), was markedly reduced.

## Discussion

Taken together, current findings promote a notion that higher levels of AAT either due to the increased intracellular expression or external entry can prevent cancer cell death. In fact, intracellular entry of AAT occurs constitutively in all mammalian cells, including cancer cells. The intracellular endocytosis of AAT may depend on the pathways non-involving lipid rafts, namely clathrin-mediated endocytosis, or pathways that take place in lipid rafts, which include caveolae-mediated endocytosis and flotillin-dependent endocytosis (91, 117, 118) (**Figure 1**). We and other investigators have previously found that cellular uptake of AAT takes place in lipid rafts (108, 118). We hypothesize that the uptake and subcellular trafficking of AAT might strongly depend on its concentration and the activation status of the cells. The uptake of high concentrations of AAT may involve clathrin-mediated endocytosis whereas lower concentrations of AAT may enter *via* caveolae pathway. Caveolae, in contrast to clathrin-coated pits, are very heterogeneous and the alterations in caveolae are not only important in tumor heterogeneity but also have a prognostic value (119). Hence, to better understand the role of AAT in tumorigenesis, intracellular entry and processing of AAT, but also other APPs, by cancer cells cannot be denied and warrants more detailed investigations.

Finally, it is important to keep in mind that AAT can modulate activities of different cells acting within tumor microenvironment. For example, fibroblasts are one of the important components of the tumor microenvironment, which participate in remodeling and a crosstalk between cancer cells and infiltrating leukocytes (120). Previously, it has been reported that AAT stimulates fibroblast proliferation and extracellular matrix production (120, 121). Remarkably, decreased p62 expression seems to be crucial for myofibroblast differentiation to support fibrosis and tumor growth (122–124). In line, findings by Schwarz and co-authors show that AAT suppresses p62 levels in lung cancer cells. AAT is also reported to affect leukocyte profiles associated with inflammatory resolution and tissue regeneration (125, 126) and to promote macrophage polarization towards the pro-tumorigenic M2-like profile (126, 127). Unfortunately, the possible influence of AAT on the interactions between tumor and tumor-associated cells, which are of critical importance in tumorigenesis, has been only minimally addressed.

Based on the current knowledge, one can conclude that the involvement of AAT as well as other APPs in cancers can be direct (on cancer cells) or indirect (*via* cancer associated cells), and dependent on protein concentration, molecular form and tumor microenvironment. APPs may form pro- or anti-cancer host defense response, which needs to be taken into consideration for designing cancer treatments. Future work will have to determine whether some of the APPs contribute to and/or reflect cancer development, therefore representing potential therapeutic targets and/or biomarkers in tumorigenesis.

## Author Contributions

SJ and SG wrote the first draft. SW prepared figure and table and helped with manuscript preparation. AG, HG, and TW added comments. All authors contributed to the article and approved the submitted version.

## Funding

This work has been supported by funding from the German Center for Lung Research (grant 82DZL002A1). 

## Conflict of Interest

The authors declare that the research was conducted in the absence of any commercial or financial relationships that could be construed as a potential conflict of interest.
